# Hi-C, a chromatin 3D structure technique advancing the functional genomics of immune cells

**DOI:** 10.3389/fgene.2024.1377238

**Published:** 2024-03-22

**Authors:** Ran Liu, Ruitang Xu, Siyu Yan, Peiyu Li, Changteng Jia, Haoqiang Sun, Kaiwen Sheng, Yongjie Wang, Qi Zhang, Jiao Guo, Xiangzheng Xin, Xinlan Li, Dianhao Guo

**Affiliations:** School of Clinical and Basic Medical Sciences, Shandong First Medical University and Shandong Academy of Medical Sciences, Jinan, Shandong, China

**Keywords:** 3D chromatin structure, Hi-C, immune cells, regulation of gene expression, diseases

## Abstract

The functional performance of immune cells relies on a complex transcriptional regulatory network. The three-dimensional structure of chromatin can affect chromatin status and gene expression patterns, and plays an important regulatory role in gene transcription. Currently available techniques for studying chromatin spatial structure include chromatin conformation capture techniques and their derivatives, chromatin accessibility sequencing techniques, and others. Additionally, the recently emerged deep learning technology can be utilized as a tool to enhance the analysis of data. In this review, we elucidate the definition and significance of the three-dimensional chromatin structure, summarize the technologies available for studying it, and describe the research progress on the chromatin spatial structure of dendritic cells, macrophages, T cells, B cells, and neutrophils.

## Introduction

### Chromatin 3D structure

The 3D chromatin structure is essential for gene regulation and cellular function. Studying the three-dimensional structure of chromatin can help us understand the spatial relationship between distal regulatory elements and target genes (Ling et al., 2022), including how the protein factors associated with enhancers [Enhancers are non-coding sequences in the genome that can activate the expression of target genes transcribed by RNA polymerase II (RNAPII) (Panigrahi and O'Malley, 2021)] promote chromatin interactions, the effect of enhancer-promoter interaction on gene expression, and the importance of histone modifications and transcription factors in the regulation of chromatin accessibility. Therefore, in-depth research on the three-dimensional structure of chromatin can reveal the mechanism of gene expression regulation, thereby pinpointing disease genes that are candidates for novel therapeutics at the genome spatial level.

**FIGURE 1:**
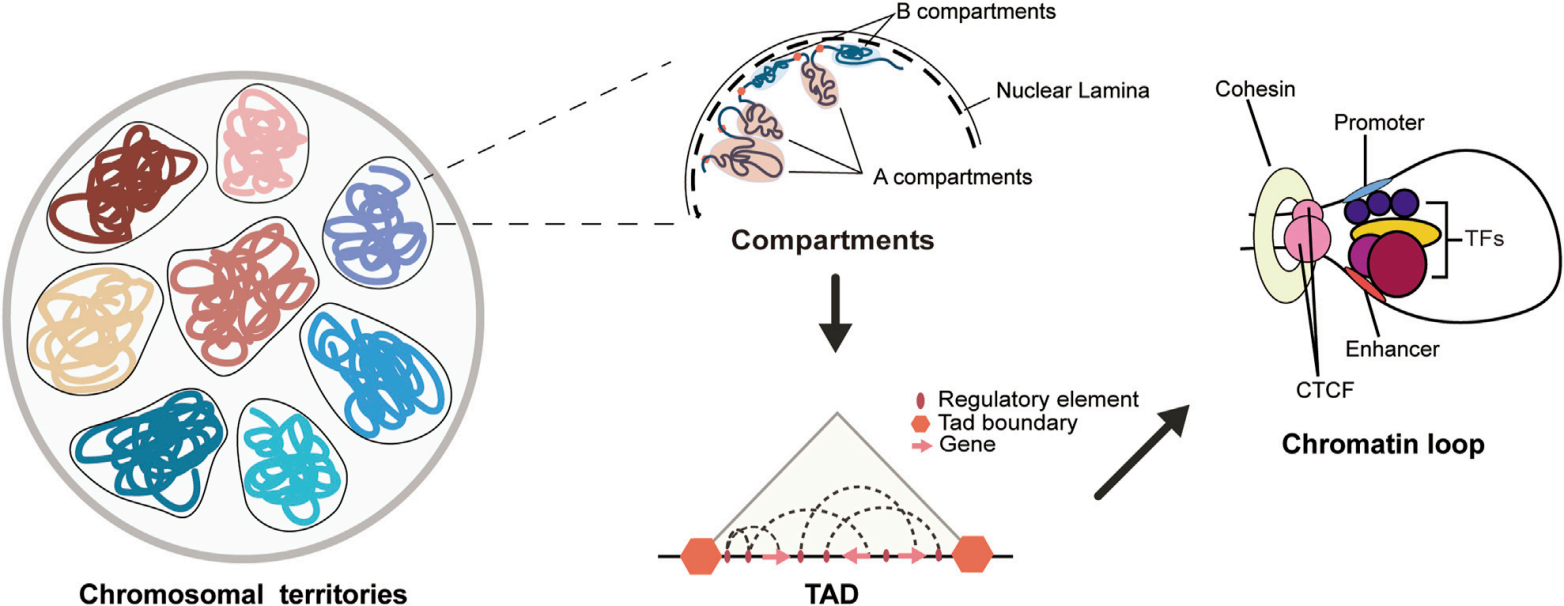
Chromatin 3D structure diagram. The 3D structure of the genome in the cell nucleus can be hierarchically organized into chromosomal territory, A/B compartments, TADs, and chromatin loops. Each chromatin domain represents a non-overlapping and independent region of each chromosome. The A/B compartments are mainly composed of A and B compartments, where the A compartment has a more open chromatin state with higher gene activity, while the B compartment has a more compact chromatin state with lower gene activity. Topologically associating domains (TADs) serve as the basic structural and regulatory units of the genome, and are enriched with various regulatory elements and their target genes. Chromatin loops are typically formed by long-range interactions between promoters and enhancers, and are regulated by the binding of histones and transcription factors to the loop, with the maintenance of the loop often involving the cooperative action of CTCF and cohesin.

The 3D chromatin structure refers to the spatial organization formed by the DNA and proteins in the nucleus of the genome. The genome is divided into chromosomal territories, A/B compartments, topologically associating domains (TADs) and chromatin loops through the three-dimensional folding of chromosomes (Figure 1) (Deng et al., 2022). Chromosomal territories refer to the distinct regions occupied by different chromosomes within the cell nucleus, which are often independent and non-overlapping (Cremer et al., 1993). According to the level of transcriptional activity, chromatin is divided into A/B compartments. The A compartment mainly consists of open chromatin, with significantly higher levels of activating histone modifications and transcriptional activity compared to the B compartment (Mohanta et al., 2021). Additionally, the I compartment (Vilarrasa-Blasi et al., 2021; Cuartero et al., 2023) is a highly dynamic intermediate compartment independent of the A and B compartments, and is rich in chromatin structures with stable promoters and repressive chromatin, capable of interacting with the A and B compartments. The compartments contain chromatin interaction domains formed by loop extrusion, known as TADs (Dixon et al., 2012; Rajderkar et al., 2023), and TADs are conserved across species and cell types (Winick-Ng et al., 2021). The boundaries of TADs are primarily maintained by CCCTC-binding factor (CTCF), condensin, cohesin, and histone modifications to ensure stability (Rajderkar et al., 2023). CTCF is a transcriptional regulatory protein that is widely present in eukaryotes. It plays an important role in maintaining the insulation of TAD boundaries. Cohesin can cooperate with CTCF to participate in the formation and regulation of chromatin loops, and it can also regulate the binding and functional effects of CTCF on chromatin (Li et al., 2020). TADs are the fundamental structural units of chromatin and the basic regulatory units of the genome, and they are enriched with various regulatory elements and their target genes internally (Lupianez et al., 2016; Barrington et al., 2019; Wang et al., 2022). New research has found that DNA double-strand breaks damage certain TADs, and these damaged TADs aggregate to form a new chromatin compartment-D compartment, which is targeted by active transcription protein markers within other undamaged TADs (Arnould et al., 2023). Chromatin loops are ring-like structures formed by proteins and other molecules mediating, folding, and encapsulating chromatin. Chromatin loops can facilitate the spatial binding [This binding may be dynamic (Xiao et al., 2021; Zuin et al., 2022)] of promoters and distal regulatory elements, such as enhancers, to regulate gene expression levels (Figure 1) (Mohanta et al., 2021). In addition, long-range loop interactions between promoters and enhancers are common means of regulating gene expression levels, and their activity is associated with a specific combination of colocalization of post-translational histone modifications, also known as “chromatin state.” Current research indicates that active promoters are enriched with H3K27ac and H3K4me2/3, while active enhancers are enriched with H3K27ac and H3K4me1/2 (Miko et al., 2021; Guo and Wang, 2022). In addition, Schoenfelder and Fraser proposed the “selecting–facilitating–specifying” model of chromatin in 2019, which provides a more dynamic explanation for the interactions between enhancers and promoters. This model involves two layers of regulatory processes. The first layer involves the activity of molecules such as transcription factors, which select cell-specific regulatory elements by increasing the accessibility of chromatin regions and depositing marks. The second layer involves compartments, TADs, and loops that bring spatial proximity to regulatory elements with similar activity states and facilitate the contact between enhancers and promoters. These two layers of regulatory mechanisms work together to ensure the specificity and stability of gene expression within the cell (Schoenfelder and Fraser, 2019).

The above classifications of chromatin 3D structure were identified using techniques such as Hi-C. In addition, researchers have also used DamID (Dam identification) or chromatin immunoprecipitation (ChIP) techniques to identify genomic regions that closely interact with the nuclear lamina (NL), known as lamina-associated domains (LADs) (van Steensel and Belmont, 2017). Through comparison and identification, LADs correspond to the B compartment, while inter-LADs correspond to the A compartment. Furthermore, LADs and TADs are very similar, and both contribute to the organization of chromatin’s topological structure (Briand and Collas, 2020). Recent studies have redefined chromatin functional units, namely RAMs, through chromatin immunoprecipitation assays. RAMs reflect the modular organization of the 3D genome and are the functional units of chromatin spatial organization (Zheng and Wang, 2022). Compared to the TADs defined by Hi-C technology, RAMs have a higher proportion of promoter-enhancer clusters, loop anchors, and super-enhancer [Super-enhancers are regulatory regions on chromatin with unusually strong enrichment for the binding of transcriptional co-activators (Pott and Lieb, 2015)] clusters. In addition, the RAMs boundary has high inhibitory histone modifications, such as H3K9me3 and H3K27me3, and the disruption of the RAMs boundary may be closely related to the occurrence of cancer (Zheng and Wang, 2022).

The 3D chromatin structure plays an important role in the regulation of gene expression, cell differentiation and disease progression (Zheng and Xie, 2019). In terms of transcriptional regulation, the formation of chromatin loops and interactions within chromatin regions ensure the proximity of regulatory elements to genes. The formation of compartments and TADs ensures the precision of gene regulation. In terms of genetic stability, the spatial organization of chromatin, particularly the formation of TAD boundaries, to some extent can prevent the occurrence of genetic abnormalities (Hsieh et al., 2020; Zhao et al., 2023). The deletion, inversion, duplication, or misalignment of TAD boundaries will affect its insulation degree and connectivity (Barrington et al., 2019; Watt et al., 2021), and may change the genome structure, so that the enhancers adjacent to TAD will ectopically activate genes, leading to gene misexpression and disease. Congenital limb malformations such as syndactyly, brachydactyly, polydactyly, Liebenberg syndrome, and autosomal dominant adult-onset demyelinating leukodystrophy (ADLD) are all associated with the loss or misregulation of TAD boundaries (Lupianez et al., 2016). Research on the pathogenesis of dilated cardiomyopathy (DCM) indicates that the overexpression of the transcription factor HAND1 leads to a significant increase in enhancer-promoter interactions, causing widespread chromatin reprogramming, ultimately resulting in transcriptional dysregulation and the onset of DCM (Feng et al., 2022). Additionally, in acute myeloid leukemia (AML), the loss of CTCF binding sites induced by high methylation leads to the loss of TAD insulation, enhancing chromatin interactions in AML, which may be associated with AML induction (Xu et al., 2022). Research on pancreatic tumors has found that the chromatin accessibility is increased near the active genes of mutated kras cells in malignant tumors, significantly increasing their epigenetic plasticity and driving tumorigenesis (Burdziak et al., 2023). Researchers have also observed the reorganization of the A, B, and I compartment in samples of colorectal cancer. These topological changes suppress the tumor’s stemness and invasive programs while inducing the expression of anti-tumor immune genes, thus inhibiting malignant tumor progression (Johnstone et al., 2020).

### Advances in 3D chromatin structure technology

At present, there are a variety of methods to study the 3D chromatin structure, and they are also developing and improving. The earliest technology used by researchers was DNA FISH (Price, 1993), which allows for the direct detection of interactions between a small number of loci within individual cells. This technology is currently developing rapidly, and when combined with super-resolution microscopy, the resolution is greatly improved. Additionally, the probe design and signal intensity have been enhanced. However, the standardization of this technology is relatively poor, and image analysis requires experienced and highly skilled personnel (Wagner and Haider, 2012; Prudent and Raoult, 2019). Later, with the continuous advancement of technology, 3C (Chromosome Conformation Capture), Capture-C, 4C (Circular Chromosome Conformation Capture) and 5C (Chromosome Conformation Capture Carbon Copy) technologies have emerged one after another.

3C (Dekker, 2006) is a technique used to detect the interaction frequency between different DNA fragments in chromatin. But the 3C technology requires rigorous operation to obtain correct 3C data. The three quality controls in the middle include: PCR efficiency, assessing the level of background random collisions, and data normalization (Dekker, 2006). Moreover, when the 3C technology is applied, the detection of the interaction fragment is limited to specific genomic intervals. In order to screen candidate fragments for interaction with target fragments without preference on a genome-wide scale, the 4C technique was developed by researchers (Simonis et al., 2006). 4C technique uses specific primers to enrich DNA fragments that interact with the target locus, enabling the study of interactions between a specific locus and the rest of the genome (Simonis et al., 2006; Göndör et al., 2008). Subsequently, researchers developed the 5C technique based on 3C, which expanded the scope of 3C applications and can be used to map the cis and trans interactions of large-scale genomic elements, as well as for deeper studies of chromosomal structures (Dostie et al., 2006). In this context, Hi-C technology came into being in 2009 (Lieberman-Aiden et al., 2009) and has become an advanced technology for studying the 3D chromatin structure, and has been widely used. Although the techniques in the 3C family are continually being upgraded and improved, their core methodological steps remain the same: crosslinking, restriction digest, and proximity ligation (McCord et al., 2020).

Furthermore, the interaction between enhancers and promoters is crucial for gene regulation. In addition to chromatin contacts, open chromatin regions can also facilitate this interaction (Bendl et al., 2022). Chromatin accessibility techniques and methods to study protein-chromatin interactions are of great significance for studying the spatial 3D structure of chromatin. High-throughput chromatin accessibility sequencing technology has become an important tool for studying open chromatin regions, such as ATAC-seq, DNase-seq, MNase-seq, scATAC-seq, etc. Among them, ATAC-seq can efficiently label and sequence open regions on chromatin by utilizing the action of Tn5 transposase (Grandi et al., 2022). DNase-seq uses DNase I, an endonuclease, to cleave DNA sequences and obtain information about open regions of chromatin through sequencing analysis (Lv et al., 2023). MNase-seq, similar to DNase-seq, uses MNase, an enzyme, to obtain chromatin accessibility information. In addition, ATAC-seq combined with single-cell sequencing technology gave birth to scATAC-seq, which is characterized by the determination of chromatin accessibility and genomic sequences within a single cell. The biggest difference between this technique and conventional ATAC-seq is that individual cells need to be labeled to ensure specificity for each cell (Wang et al., 2023). Techniques for identifying specific protein binding sites include ChIP-seq and CUT&Tag. ChIP-seq is a genome-wide high-throughput sequencing technique to study the binding of proteins to target DNA sequences, providing a method for epigenetic studies such as transcription factors and histone modifications (Nakato and Sakata, 2021). CUT&Tag is a novel technology that has been improved based on ChIP-seq, significantly enhancing the signal-to-noise ratio of histone marks by utilizing the activity of Tn5 transposase, while also offering the advantages of low cost and high efficiency (Kaya-Okur et al., 2020). Of particular note, CUT&Tag technology has the capability to handle low-cell-number samples, thus holding broad potential for applications in single-cell chromatin analysis, offering a new avenue for studying chromatin organization. In addition, the ABC computational model can be used to calculate the contact frequency between cell-specific gene promoters and enhancers, and can predict the connections between enhancers and genes (Fulco et al., 2019; Nasser et al., 2021). The above-mentioned tools can be used to reveal mechanisms of changes in gene expression levels. Additionally, direct techniques for detecting gene expression levels include RNA-seq (Wang et al., 2009), among others.

**FIGURE 2:**
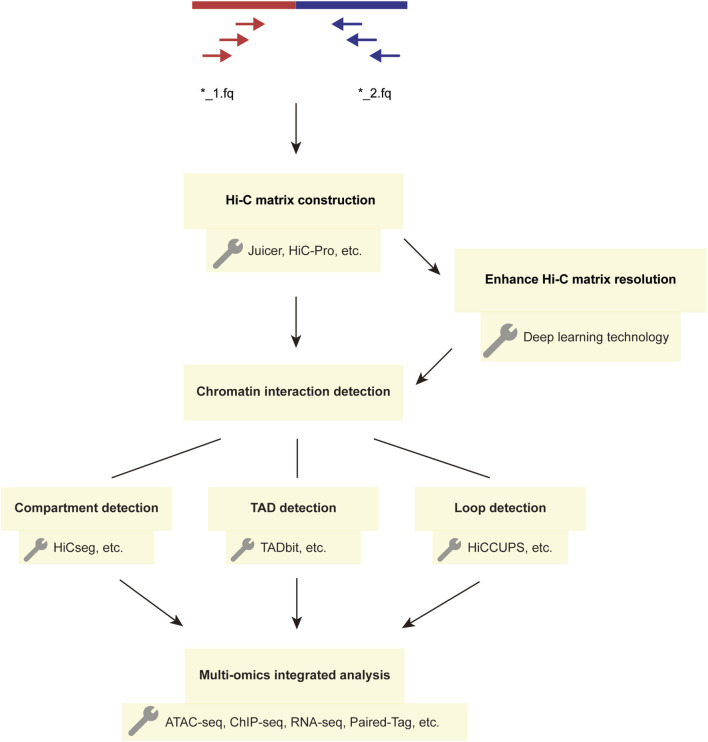
The Workflow and Techniques Used in Hi-C Data Analysis. The main steps in the Hi-C analysis workflow involve constructing matrices and detecting chromatin interactions using the paired-end sequencing data obtained from two FASTQ files. The detected chromatin structural variations can be further analyzed using multi-omics methods, and deep learning techniques can enhance Hi-C interaction matrices to improve matrix resolution.

Furthermore, recently developed single-cell multi-omics detection methods, such as Paired-Tag and scREG (Zhu et al., 2021; Duren et al., 2022), have emerged. Both of these techniques enable integrated analysis of the transcriptome and chromatin accessibility within single cells (Figure 2).

### Hi-C and its derivative technologies

Hi-C is a technology based on 3C and utilizes high-throughput sequencing to detect chromatin interactions across the entire genome (Lafontaine et al., 2021). Hi-C technology can unveil the intricate folding structure of chromatin, which is closely linked to the proportion of cells in a population that experience specific contacts. Additionally, this method allows for the study of interactions within chromatin at a nanometer-scale distance. However, Hi-C is limited in its ability to determine only the relative distance and interaction frequency between different regions on the chromosome. As a result, the data can only represent the relative contact frequency between these regions. Furthermore, the lack of standardization within Hi-C hinders the comparison of data across different conditions and cell types. At the same time, the standard Hi-C method can only be used to detect the interaction between two specific regions on the chromosome, and cannot reveal whether multiple regions simultaneously or mutually repel each other (McCord et al., 2020).

Hi-C includes a biotinylation step between digestion and ligation, giving Hi-C a better ability to enrich for ligated junctions compared to 3C, making it more favorable for the analysis of chromosomal spatial structure. In 2012, the DNase Hi-C technology was developed (Duan, 2021). Compared to Hi-C, the major improvement of this technique lies in the use of DNase I for chromatin cleavage, overcoming the limitations of low digestion efficiency and accuracy associated with the traditional Hi-C method (Ma et al., 2015). In 2013, Micro-C was proposed as a complementary method to Hi-C. This technique uses micrococcal nuclease instead of the restriction enzyme used in Hi-C to fragment chromatin, and is suitable for short-range analysis of nucleosome fiber folding (Hsieh et al., 2015). In 2013, single-cell Hi-C was used, which is able to visualize the three-dimensional structure of chromatin within a single cell with specificity (Nagano et al., 2013). However, this technique cannot reveal the interactions between each restriction fragment and all of its spatially proximal loci, as bulk Hi-C does (Nagano et al., 2013). This problem can be addressed by the Multiplex-GAM technology mentioned later in the text (Beagrie et al., 2023). In 2014, the concept of *In situ* Hi-C was proposed, which reduces false positive results, improves the signal-to-noise ratio, and shortens the experimental cycle compared with Hi-C (Rao et al., 2014). In 2017, DLO Hi-C technology was developed. Compared to Hi-C, this technique offers the advantages of lower cost, simplified library construction, shorter processing time, and higher yield of effective data (Zhang et al., 2020a). Also in 2017, Hi-C 3.0 was utilized (Akgol Oksuz et al., 2021). Compared to Hi-C, this technique offers higher resolution and improved reproducibility, allowing for the detection of thousands of loops. In addition, Capture-C is also a high-resolution technique based on 3C for detecting chromatin interactions, which allows for the extraction of restriction fragments of interest from *in situ* 3C material, such as promoters, enhancers, and super-enhancers (Downes et al., 2022). The emergence of these emerging technologies enables scientists to identify interaction patterns between different genes, transcription factors, and regulatory elements, uncover key genes or transcription factors, and further interpret gene function (Zhang et al., 2013).

In recent years, many new studies have demonstrated further optimized techniques related to chromatin conformation capture. Micro-Capture-C (MCC) is a technique that combines Micro-C with Capture-C, with high resolution that allows precise localization to individual base pairs (Crump et al., 2023). Region Capture Micro-C (RCMC) is a method that combines region capture with Micro-C, which resolves highly nested and focal interactions, most of which are nested interactions between promoter and enhancer regions, and labels these interactions as “microcompartments.” Additionally, RCMC technology can mine sequencing-limited data to generate high-depth 3D genome maps (Goel et al., 2023). Multiplex-GAM is a technology used for genome-wide interaction analysis, capable of detecting multiple genomic regions missed by Hi-C (Beagrie et al., 2023). Moreover, this technology can provide a comprehensive understanding of the 3D chromatin structure at the single-cell level (Beagrie et al., 2023). Pore-C, a chromatin conformation-capturing technique combined with nanopore sequencing of linkers, enables faster analysis of proximal higher-order chromatin contacts at the genome scale (Deshpande et al., 2022). Compared to Hi-C, which can only detect interactions between paired genomic loci, this technique can detect interactions between two or more DNA loci by using long-read sequencing (Deshpande et al., 2022).

HiChiP is a technique that combines Hi-C and chromatin immunoprecipitation (ChIP), allowing for the simultaneous detection of chromatin interactions and protein binding sites on chromatin. Specifically, H3K27ac HiChIP can detect interactions at higher resolution within promoters and enhancers (Mumbach et al., 2017). Promoter capture Hi-C (PCHi-C) is a derivative technique primarily used to identify the interaction regions between enhancers and promoters. It is of significant importance for studying the relationship between genome regulatory mechanisms and disease occurrence. When this technology is used in conjunction with expression quantitative trait loci (eQTL) analysis, it can better reveal the relationship between disease gene expression and chromosomal spatial structure (Javierre et al., 2016; Baxter et al., 2018).

### Analysis workflow of Hi-C data and deep learning techniques

The analysis process of Hi-C data typically includes the following steps (Ay and Noble, 2015): first, the established Hi-C library is subjected to quality control filtering to obtain high-quality data. During the data processing, the data is aligned to the desired genome following the principle of paired-end unique mapping, and an interaction matrix is generated. Subsequently, the matrix is subjected to normalization, followed by the detection of chromatin interactions. Finally, the data is visualized for further analysis (Figure 2). In constructing the Hi-C matrix, we typically use two tools, Juicer (Durand et al., 2016) and HiC-Pro (Servant et al., 2015). Both Juicer and HiC-Pro can directly convert raw data into normalized contact maps (Servant et al., 2015; Durand et al., 2016). Juicer can automatically annotate the three-dimensional structure of chromatin, while HiC-Pro can construct allele-specific contact maps using phased genotype data (Servant et al., 2015; Durand et al., 2016). When analyzing chromatin structure, a series of tools are typically used to detect structures at different levels. For compartment detection, tools such as HiCSeg (Lévy-Leduc et al., 2014) and Fit-Hi-C2 (Kaul et al., 2020) are employed. For TAD detection, we use tools such as TADbit (Serra et al., 2017) and TADpole (Soler-Vila et al., 2020). The most significant feature of TADbit is its ability to complete the entire process of Hi-C data processing and analysis. It is important to note that when analyzing interaction matrices, this method uses a breakpoint detection algorithm to calculate the position of TADs along chromosome boundaries and employs the Needleman-Wunsch algorithm to align TADs (Serra et al., 2017). TADpole’s advantage lies in its ability to identify hierarchical structural domains in interaction matrices and robustness in terms of data resolution, normalization strategy, and sequencing depth (Soler-Vila et al., 2020). For the detection of chromatin loops, we often use tools such as HiCCUPS (Durand et al., 2016), GOTHiC (Mifsud et al., 2017), and HiCExplorer (Wolff et al., 2022). In fact, the scope of these tools is not absolute; some tools can detect multiple chromatin substructures. Table 1 provides a comparison of the prominent features of these analysis tools.

**TABLE 1:** Analysis tools for Hi-C data.

Analysis tools	Features	References
Juicer	A fully automated pipeline for processing and annotating data from Hi-C and other contact mapping experiments	Durand et al. (2016)
HiC-Pro	Ability to use staged genotypic data to construct allele-specific contact profiles	Servant et al. (2015)
HiCSeg	Two-dimensional segmentation of Hi-C data for the detection of homeopathic interaction regions	Lévy-Leduc et al. (2014)
Fit-Hi-C2	Suitable for studying medium-range chromatin interactions without any parametric assumptions	Kaul et al. (2020)
TADbit	Ability to complete the entire process of Hi-C data processing, analysis, and visualization	Serra et al. (2017)
TADpole	Ability to study hierarchical layers of the Hi-C matrix	Soler-Vila et al. (2020)
HiCCUPS	An algorithm of Juicer, used to identify chromatin loops	Durand et al. (2016)
GOTHiC	A binomial probabilistic model that solves the complex bias of Hi-C raw data and distinguishes between true and false interactions	Mifsud et al. (2017)
HiCExplorer	Ability to detect TADs and loops with high detection rate and accuracy	Wolff et al. (2022)

In addition, deep learning techniques have been applied to computational 3D genomics. Deep learning is a machine learning method that simulates the structure and function of the human brain’s neural network, and its greatest feature is “learning from what is seen” (Li et al., 2023). Based on the amount of labeled data used for training, deep learning can be divided into three modes: supervised learning, unsupervised learning, and semi-supervised learning. Supervised learning uses all labeled data as the training set, and the trained model can predict the remaining unlabeled data. Convolutional Neural Network (CNN) is an example of this learning mode. Unsupervised learning uses unlabeled data as the training set and aims to discover hidden information in the data, as well as to perform functions such as dimensionality reduction, clustering, and annotation. Autoencoders, Generative Adversarial Networks (GAN), and self-supervised learning are examples of unsupervised learning. Semi-supervised learning involves training with a small amount of labeled data and a large amount of unlabeled data, resulting in a model with enhanced performance and generalization capabilities (Srinidhi et al., 2021; Chen et al., 2022). Currently, deep learning has been applied in various fields, and in the study of chromatin three-dimensional structure, it has primarily been used to enhance the resolution of Hi-C matrices and to predict 3D spatial structures, including compartments, TADs, and chromatin loops, using high-resolution matrices. The deep learning techniques for enhancing the resolution of Hi-C data include HiCPlus (Zhang et al., 2018), HiCNN [Liu and Wang (2019a)], HiCNN2 [Liu and Wang (2019b)], SRHiC [Li and Dai (2020)], VEHiCLE [Highsmith and Cheng (2021)]. The high-resolution matrix prediction models based on GAN include hicGAN (Liu et al., 2019), DeepHiC (Hong et al., 2020), EnHiC [Hu and Ma (2021)], HiCARN [Hicks and Oluwadare (2022)]. Other deep learning models include iEnhance (Li et al., 2023) and Orca (Zhou, 2022). The specific content is as shown in Table 2.

**TABLE 2:** Deep learning techniques.

Deep learning techniques for enhancing Hi-C data resolution	Features	References
HiCPlus	Train a 3-layer deep convolutional neural network (ConvNet) and use only 1/16 of the sequencing read length to construct interaction matrices of similar quality	Zhang et al. (2018)
HiCNN	Training a 54-layer deep convolutional neural network, the training results are superior to the high-resolution Hi-C data replicated by HiCPlus, but the training time is longer	Liu and Wang (2019a)
HiCNN2	Use three different levels of deep convolutional neural networks, with 56 layers, 22 layers, and 3 layers respectively. It outperforms HiCNN and HiCPlus in predicting high-resolution Hi-C contacts and recovering important genomic interactions	Liu and Wang (2019b)
SRHiC	Train multiple times to use small convolutional neural networks and infer corresponding high-resolution Hi-C interaction matrices from low-resolution subsampling ratios of 1/16 and 1/25, further reducing sequencing costs. It outperforms HiCPlus and HiCNN in the identification of long-range interactions and TAD boundary detection	Li and Dai (2020)
VEHiCLE	Use a deep variational autoencoder network and excel in improving the accuracy of insulation scores. It is particularly meaningful for studying the chromosomal structure at specific genomic locations	Highsmith and Cheng (2021)
High-resolution matrix prediction model based on GAN		
hicGAN	Be the first technique to apply GAN to generate 3D genomic data and capable of predicting chromatin interactions in low-coverage Hi-C data	Liu et al. (2019)
DeepHiC	Using conditional generative adversarial networks (cGAN), it is possible to reproduce high-resolution Hi-C data from subsampled reads as low as 1%	Hong et al. (2020)
EnHiC	It outperforms hicGAN and DeepHiC, providing more accurate predictions of TADs and fine chromatin interactions	Hu and Ma (2021)
HiCARN	Use two cascaded residual networks: a convolutional neural network and a generative adversarial network	Hicks and Oluwadare (2022)
Other deep learning models		
iEnhance	A chromatin interaction enhancement network that integrates multi-scale spatial projection, attention fusion, dense channel encoding, and residual channel decoding architecture, is capable of enhancing low-resolution Hi-C matrices. Specifically, it excels in enhancing A/B compartments (with a stronger effect on the A compartment than the B compartment), reconstructing small TADs, and recovering subtle chromatin loops	Li et al. (2023a)
Orca	A prediction model consisting of a hierarchical sequence encoder from convolutional networks and multi-level cascaded decoders, is capable of predicting chromatin 3D structures from kilobase to whole chromosome scales	Zhou (2022)

### Applications of Hi-C

The Hi-C technology is a method for studying chromatin structure, which can be used to investigate the relationship between chromatin structure changes and gene expression. It can be applied to study gene regulation and the mechanisms of disease occurrence (van Berkum et al., 2010). In terms of cell differentiation, scientists have studied the interaction patterns between enhancers and promoters across the entire genome during the differentiation process of human primary keratinocytes (Gong et al., 2021). In the context of cancer, researchers have compared the TAD boundaries and quantities between myeloma cells and normal cells (Gong et al., 2021). In mammalian embryonic development, scientists have investigated the strength of chromatin interactions between mammalian sperm and egg cells at various stages of division (Ke et al., 2017). Furthermore, scientists have used Hi-C to accurately locate the centromeres in the yeast genome (Varoquaux et al., 2015). With the continuous development of Hi-C technology, it is gradually gaining importance in various fields such as agricultural science, life science, and medical science.

In addition, researchers can gain in-depth understanding of the spatial organization and interaction patterns of the genome in immune cells through the use of Hi-C and other high-throughput sequencing technologies, thereby revealing the complex network of gene regulation. Hi-C can reveal interactions between elements on the chromatin loop in immune cells, interactions of genes within TADs, as well as interactions between compartments, among others. Research on the spatial organization and interactions of the genome is crucial for unraveling the functionality of the immune system and the mechanisms underlying related diseases. Next, we will further explore the chromatin 3D structure of immune cells and important findings related to immune system functionality and disease mechanisms.

### The 3D chromatin structure of immune cells

#### Dendritic cells

Classical dendritic cells (cDCs) complete their differentiation process in the bone marrow as important antigen-presenting cells in the immune system. The differentiation process is mainly undergoing hematopoietic stem cells (HSCs), lymphosensitized pluripotent progenitor cells (LMPPs), common DC progenitors (cDPs), and classical dendritic cells type 1 (cDC1s) or classical dendritic cells type 2 (cDC2s) (Kurotaki et al., 2022). The research found that the IRF8 factor promotes the enrichment of H3K27ac, leading to chromatin activation, thereby promoting the formation of the A compartment in cDC cells and activating the expression of specific genes (Kurotaki et al., 2022). Furthermore, the study also found that in cDC cells not infected by intracellular pathogens such as Toxoplasma gondii, the chromatin 3D structure related to host defense genes (such as genes encoding cytokines and chemokines) has already been established (Kurotaki et al., 2022), with the most significant being the formation of TAD loops and the strengthening of interactions within TADs, which provides a pre-existing framework for the action of the aforementioned IRF8 factor. During the differentiation process from common DC progenitors (cDPs) to classical dendritic cells (cDCs), the internal interactions within TADs are strengthened, and TAD loops are reinforced. These factors promote the transition of cDC-specific genes from the B compartment to the A compartment.

Classical dendritic cells (cDCs) play a key role in the recognition of pathogens and innate immune responses. Even before infection, the three-dimensional structure of higher-order chromatin in cDC cells involved in immune defense is established, which can generate a faster and stronger immune response (Kurotaki et al., 2022).

#### Macrophages

Macrophages can differentiate from monocytes in response to local tissue growth factors or certain pathogenic microorganisms. During the differentiation process from progenitor cells to macrophages, regulation is mediated by the CSF1 factor and the transcription factor PU.1 (Pollard, 2009). Macrophages are the most plastic cells in the hematopoietic system, and they can polarize into two distinct phenotypes: M1 and M2. M0 macrophages can be polarized into M1 macrophages by stimuli such as polarization activators (such as TAF-α, TFN-γ, LPS, etc.) or chemokines (such as CCL2, CCL3, etc.). Research has also found that IL-26 can activate NF-κB, further promoting M1 polarization (Lin et al., 2020). M1 macrophages exhibit potent antimicrobial and antitumor activity. Conversely, M0 macrophages, when stimulated by polarization activators (such as IL-4, IL-10, and IL-13) or chemokines (such as CCL17, CCL22, etc.), can polarize into M2 macrophages (including M2a, M2b, M2c, and M2d subtypes) (Kishore and Petrek, 2021). M2 macrophages demonstrate strong phagocytic capabilities and can clear debris and apoptotic cells, thereby promoting tissue repair and wound healing (Shapouri-Moghaddam et al., 2018). The PTGS2 gene encodes cyclooxygenase (PGHS-2), and the higher the expression level of PGHS-2, the stronger the role of M1 in the inflammatory response (Wang and Zhao, 2022). In addition, genomic regions of GBP genes such as GBP1, GBP2, and GBP5 and the STAT1 gene play a promoting role in the inflammatory response and phagocytosis processes of macrophages (Qin et al., 2017; Xiong et al., 2022). In summary, macrophages play an important role in inflammation and tissue repair.

Macrophages differentiate from monocytes, and currently the THP-1 cell line (a human monocytic leukemia cell line) is commonly used by researchers as a monocyte model (Zhang et al., 2020b). In this section, we will introduce the differences in chromatin 3D architecture between this cell line and primary monocytes. Compared to primary monocytes, THP-1 cells exhibit fewer interactions between large and small chromosomes, and the interactions between small chromosomes are more pronounced. Additionally, the overlap of TAD boundaries between these two cell types is minimal, at only 25%. Correspondingly, 20% of the genome in these two cell types belongs to different A/B compartments (Zhang et al., 2020b). For example, genes involved in host defense (such as DEFA1, DEFA1B, and DEFA3) are located in the A compartment of chromatin in primary monocytes, but in the B compartment of chromatin in THP-1 cells. The expression levels of these genes are higher in primary monocytes than in THP-1 cells. Conversely, genes encoding cell cycle-related protein kinases (such as CDC7) are located in the A compartment of chromatin in THP-1 cells, but in the B compartment of chromatin in primary monocytes, and the expression levels of these genes are higher in THP-1 cells (Liu et al., 2021).

The differentiation process of macrophages reflects the importance of chromatin 3D structure. During the differentiation of monocytes into macrophages, there is an increase in the frequency of interactions within small chromosomes, while the interaction strength between large and small chromosomes significantly decreases. Furthermore, in differentiated macrophages, the interactions within TADs related to immune and inflammatory responses are stronger compared to primary monocytes. Correspondingly, the expression levels of genes within these TADs decrease, such as the PTGS2 gene, chemokine genes (CXCL1, CXCL2, and CXCL3), and chemokine receptor genes (CCR2, CCR7, and CX3R1) (Zhang et al., 2020b). Furthermore, during the differentiation process, there is an increase in TAD boundary insulation and enhanced interactions within TADs (Minderjahn et al., 2022). Moreover, in differentiated macrophages, low-affinity CTCF is removed from the chromatin loops, while high-affinity CTCF is retained, leading to an increase in chromatin loop strength. Simultaneously, the ratio of enhancer-enhancer loops in chromatin loops is greater than enhancer-promoter loops, and AP-1 positively regulates gene transcription by binding to distal regulatory elements within these chromatin loops (Phanstiel et al., 2017).

The changes in the 3D chromatin structure of macrophages play a significant role in the occurrence and development of certain diseases. After infection of macrophages (differentiated from THP-1 cells induced by PMA) with *Mycobacterium tuberculosis*, the interactions within the chromatin become more organized, and the number of chromatin loops increases. Specifically, the genomic regions of GBP genes are found to be in close proximity to super-enhancer regions (BRD4 and MED1), which enhances the expression of immune genes such as GBP (Lin et al., 2022). Furthermore, a small subset of changes in TAD boundaries affects the expression of immune-related genes (Lin et al., 2022). Specifically, the STAT1 gene is defined within an independent TAD, leading to its activation and expression, as well as an increase in the expression of chemokine genes (such as CCL1, CCL2, CCL7, and CCL8 genes). In addition, in macrophages infected with *M. tuberculosis*, NF-κB strengthens the interactions within the chromatin loops by binding to the enhancer and promoter regions of the PD-L1 gene, activating the expression of PD-L1. Therefore, the enhancer of PD-L1 can be a target for tuberculosis treatment (Lin et al., 2022). In the anti-infection response, IRF1 can increase the chromatin accessibility of ISG loci in human macrophages, thereby promoting the overall expression of ISG genes and enhancing the body’s immune response (Song et al., 2021). In sepsis, the artificial inhibition of the histone methyltransferase EZH2 activity in humans can exert an anti-inflammatory effect by reducing the aggregation of macrophages in the renal interstitium, thereby protecting the kidneys (Li et al., 2023). In prostate cancer, the development of malignant tumors is closely associated with the simultaneous enrichment of YY1 and H3K27ac ChIP-seq signals in the specific enhancer of the IL-6 gene in M2 macrophages (Chen et al., 2023). In macrophage research, scATAC-seq can be used to depict the chromatin accessibility landscape within individual cells, and clustering annotation through UMAP can be used to study the occurrence of specific pathologies in macrophages (Sun et al., 2023). In autoimmune diseases, certain patterns of the chromatin 3D structure in monocytes and macrophages are also evident (Xia et al., 2022). The occurrence of systemic lupus erythematosus (SLE) is closely related to IFN: the combination of IFN-α and TNF can increase the chromatin accessibility of tolerance genes (such as IL6), leading to the appearance of tolerant monocytes. The chromatin accessibility of tolerance genes in monocytes from SLE patients is similar to that of tolerant monocytes (Park et al., 2017). In Table 3, we have listed the changes in the chromatin spatial structure during the aforementioned diseases or response processes.

**TABLE 3:** Genomic spatial structure alterations in B cells and macrophages during disease or a certain reaction.

Cell type	Disease or a certain reaction	Genomic spatial structure alterations
Macrophage	Anti-tuberculosis infection	The super-enhancer immunogenomic GBP interaction is strong, and NF-κB binds to the enhancer and promoter loops of the PD-L1 gene
	Anti-infection response	IRF1 increases the chromatin accessibility of ISG gene loci in macrophages
	Prostate cancer	The development of malignant tumors is associated with the enrichment of YY1 and H3K27ac at the IL-6 gene enhancer site
	Systemic Lupus Erythematosus	The occurrence of the disease is related to the binding of IFN-α and TNF, which leads to the opening of tolerant gene loci, resulting in the appearance of tolerant monocytes
B cell	Diffuse large B-cell lymphoma	The inactivation of the PRDM1 sequence at the TAD boundary in B cell chromatin leads to malignant proliferation of B cells
	Mantle cell lymphoma (MCL)	The chromosomal interactions in the oncogenic region of SOX11 have significantly increased
	Chronic lymphocytic leukemia	The 3D interactions and active enhancers in the EBF1 genomic region within B cells are lost in the early stage of the disease

#### B cells

B cells play an important role in humoral immunity, and their growth process mainly involves development and activation. B cells originate from hematopoietic stem cells and undergo a developmental process in the bone marrow, progressing through the stages of early lymphoid progenitor cells, common lymphoid progenitor cells, pre-pro B cells, pro B cells, and pre B cells. (Hua and Hou, 2013; Ng et al., 2020), where IL-7 and BCR signals play important regulatory roles (Ren et al., 2022). Furthermore, when circulating in the peripheral blood, BAFFR and TLR signals promote the positive selection and maturation of B cells (Hua and Hou, 2013). Additionally, B cell activation mainly occurs in secondary lymphoid organs such as lymph nodes, where naive B cells differentiate into germinal center B cells (GCB), which then further differentiate into memory B cells and plasma cells (Azagra et al., 2020).

During the process of humoral immunity, B cells stimulated by antigens differentiate into GCB that undergo rapid proliferation and can secrete mature high-affinity antibodies. Compared to naive B cells, GCB cells exhibit a high enrichment of gene promoter DNA interactions, forming a highly interactive and specific enhancer-promoter network centered around the BCL6 gene (Bunting et al., 2016). Unlike traditional compartment classification, it makes sense that genes in B cells are divided into three compartments: A compartment, B compartment, and I compartment. Among them, the I compartment is a chromatin structure rich in stable promoters and inhibitory chromatin independent of the A and B compartments, and the I compartment is able to interact with the A and B compartments (Vilarrasa-Blasi et al., 2021). During the differentiation process of naive B cells into germinal center B cells, the majority of stable promoters and inhibitory chromatin within the I compartment transition to the A compartment (activation event), leading to the global activation and specific expression of the activation-induced cytidine deaminase (AICDA) gene. A small portion transitions to the B compartment (inactivation event), increasing the chromosome status of genes in the A and B compartments to promote the differentiation process of B cells (Vilarrasa-Blasi et al., 2021). Research on the transcription factor STAT3 through ChIP-seq has shown that STAT3 primarily regulates gene expression in B cells by modulating distal regulatory elements (Wu et al., 2022). Additionally, the ATAC-seq profile of pre-pro B cells lacking the tumor suppressor gene PTEN undergoes changes, leading to enhanced chromatin accessibility in the regions where T-lineage and myeloid-lineage transcription factors are located, thereby promoting the development of these two lineages (Xu et al., 2023).

In disease, the three-dimensional chromatin structure of B cells also undergoes changes. In the chromatin of B cells, the inactivation of the PRDM1 sequence at the TAD boundary leads to malignant proliferation of B cells, transforming them into lymphoma cells (Nagai et al., 2019). Thus, the PRDM1 sequence may serve as a drug target for future lymphoma therapy (Xia et al., 2017). Additionally, in a comparative analysis of aggressive mantle cell lymphoma (cMCL) and indolent non-nodal mantle cell lymphoma (nnMCL), researchers found a significant increase in chromosomal interactions involving the SOX11 oncogene region in cMCL, primarily concentrated on chromosome 2, playing a key role in tumor invasion (Vilarrasa-Blasi et al., 2021), providing direction for targeted therapy of MCL. Furthermore, the early compartmental switch (A to I) of the EBF1 factor in B cells of patients with chronic lymphocytic leukemia results in the loss of chromatin interactions, and therefore, the downregulation of EBF1 has become a diagnostic marker for chronic lymphocytic leukemia (Vilarrasa-Blasi et al., 2021). In Table 3, we have listed the changes in the chromatin spatial structure during the aforementioned diseases or response processes.

#### T cells

T cells mature in the thymus and become activated into various subsets of T cells in peripheral immune organs such as the spleen, including effector T cells, memory T cells, and regulatory T cells. The development process of T cells mainly involves the stages of pro-T cells, CD4^−^CD8-double-negative (DN) thymocytes, CD4^+^CD8^+^ double-positive (DP) thymocytes, CD8^+^ single-positive T cells, and CD4^+^ single-positive T cells (Kumar et al., 2018). Among them, CD8^+^ T cells are cytotoxic cells, capable of killing infected malignant cells and playing an important role in the body’s response to infections. CD4^+^ T cells are helper cells, with the main function of mediating the activity of immune cells. Under the influence of cytokines (such as IL-12, IFN-γ, etc.) and mTOR signaling, CD4^+^ T cells can differentiate into Th1, Th17, and Th2 subsets (Geltink et al., 2018). Additionally, research has found that the SATB1 factor controls the differentiation of Th1 and Th2 cells by influencing the secretion of the two cytokines IL-4 and INF-γ (Kakugawa et al., 2017). The Tox2 locus is actively modified by H3K4me2 histone in Tfh cells, and ATAC-seq analysis also indicates high chromatin accessibility of this gene. Furthermore, Tox2 promotes the differentiation of Tfh cells and increases the expression of the Bcl6 gene within Tfh cells (Xu et al., 2019). Furthermore, the specific genomic structure within memory Th2 cells enables them to rapidly mount secondary immune responses. Compared to the initial CD4^+^ T cells, resting memory Th2 cells possess specific open chromatin regions, which are primarily enhancers. These enhancers target a limited set of specific genes, and the topological chromatin connections between these genes and enhancers are increased, priming the cells for rapid transcription of genes upon activation (Onrust-van Schoonhoven et al., 2023).

In healthy human blood, CD4^+^ and CD8^+^ T cells are activated under stimulation with CD3 and CD28. The activated CD4^+^ and CD8^+^ T cells preferentially disrupt the long-range chromatin interactions, leading to enhanced short-range interactions. Additionally, in the activated state, the number of TADs in these cells becomes more numerous and smaller, the proportion of overlapping TADs increases, and the chromatin accessibility at the boundaries of overlapping TADs is elevated. Correspondingly, the expression levels of genes within these regions also increase (Bediaga et al., 2021). In summary, T cell activation is a process that reshapes TADs and chromatin interactions, with minimal impact on compartments. Factors such as Tcf1, Lef1, and SATB1 have a certain impact on the three-dimensional chromatin structure of T cells. In CD8^+^ T cells, the transcription factor Tcf1 reduces the insulation of TAD boundaries (Wang et al., 2022), so the interaction between TADs with higher levels of Tcf1 expression is more intense. Additionally, Tcf1 and Lef1 affect the chromatin loop strength in CD8^+^ T cells by binding to specific chromatin accessible regions and super-enhancers, thereby regulating gene expression. For example, in CD8^+^ T cells of individuals with Tcf1 deficiency, the interaction of chromatin segments carrying the Myb gene is significantly reduced (Wang et al., 2022). SATB1 promotes the transcriptional activation of chromatin by facilitating the interaction between promoters and super-enhancers on the chromatin (Zelenka et al., 2022). As the interaction between the Bcl6 gene promoter and super-enhancers is strong in DP cells, the expression of the Bcl6 gene is downregulated in SATB1-deficient DP thymocytes (Feng et al., 2022). In SATB1-deficient thymocytes, the average strength of all chromatin loops is slightly reduced, primarily concentrated at the loops associated with promoters, super-enhancers, and SATB1 clusters. Specifically, in SATB1-deficient DP cells, the chromatin loops associated with promoter-enhancer and super-enhancer-super-enhancer interactions become smaller (with an average loop size of 120 kb in DP cells compared to 100 kb in SATB1-deficient DP cells). SATB1 is crucial for the transition from DP to SP cells, so in individuals with SATB1 deficiency, the generation of CD8^+^ T cells is reduced, the expression level of PD-1 is increased, tumor immunity is suppressed, ultimately leading to cancer development (Zelenka and Spilianakis, 2020). Currently, the expression of SATB1 has become an adverse prognostic marker for gliomas and colorectal cancer (Zelenka and Spilianakis, 2020).

#### Neutrophils

Neutrophils originate from multipotent hematopoietic stem cells and develop from myeloblasts. It is generally believed that they undergo sequential stages including myeloblasts, promyelocytes, myelocytes, metamyelocytes, rod nuclear granulocytes, and polymorphonuclear segmented cells (Liew and Kubes, 2019). As a type of myeloid white blood cell, neutrophils are the most abundant white blood cells in human blood, accounting for approximately 50%–70% of the total peripheral blood leukocytes in adults (Liew and Kubes, 2019). Neutrophils possess phagocytic and bactericidal functions, participate in inflammatory responses, respond to chemotactic signals, and migrate to the site of infection (Mayadas et al., 2014; Liew and Kubes, 2019).

Unlike other immune cells, neutrophils have a large number of interchromosomal interactions within the cell nucleus, but the interchromosomal gene regulation is rarely observed in this nucleus (Keenan et al., 2021). In addition, compared to progenitor cells, this cell nucleus has a large number of long-range genomic interactions, including large genomic distances between compartments A and B (Zhu et al., 2017).

In the transcriptional regulation of neutrophils and related immune diseases, the binding and variation of the regulatory factor PU.1 are associated with the local chromatin state, physical interactions between enhancers and promoters, and the coordinated expression of downstream genes (Watt et al., 2021). In neutrophils, the transcription factor C/EBPβ is continuously expressed during development and upregulated during its terminal differentiation process, while PU.1 can bind to it in a repressive chromatin state and activate it by recruiting other factors (Watt et al., 2021). PCHi-C data indicates that the enrichment of PIR (Promoter Interacting Regions) within neutrophil-specific PU.1 binding sites and their enhancers is positively correlated with the expression levels of the genes they contact. In terms of genetic effects, the impact of PU.1 tfQTLs (transcription factor quantitative trait loci) on C/EBPβ binding decreases with increasing distance, while there is no distance dependency in the binding to CTCF, indicating long-range genetic effects of CTCF binding (Watt et al., 2021). Additionally, comparative analysis of PCHi-C data from neutrophils and monocytes reveals that enhancers shared in neutrophils exhibit stronger genetic effects, and the specific binding of PU.1 to C/EBPβ in neutrophils is also associated with their specific chromatin activity (Watt et al., 2021), demonstrating that distal PU.1 binding is cell-type specific, and that this gene-determined PU.1 binding difference is related to the chromatin states of different tissues. Another study using an *in vitro* migration modelfound that after neutrophils undergo contraction migration, the overall short-range contacts between genomic regions are reduced or even interrupted. Additionally, it was observed that TADs and compartments are enriched in inactive chromatin, with a more pronounced disruption in compartment B compared to compartment A. This suggests that during the migration of neutrophils, local disruptions primarily occur in inactive chromatin, while transcriptionally active DNA remains unaffected by reshaping (Jacobson et al., 2018).

## Conclusion

The three-dimensional chromatin structure within immune cells is complex and variable, and our article discusses a topic that is currently receiving significant attention in the field of immunology. We summarized the changes in chromatin loops, TADs, and A/B compartments, as well as the effects of various cis-regulatory elements and transcription factors on the genome’s 3D structure during the differentiation and development processes of dendritic cells, macrophages, B cells, T cells, and neutrophils. In dendritic cells, we focused on the impact of the IRF8 factor on intra-cellular compartments and TADs. In macrophages, we described the changes in chromosomal interactions and intra-TAD interactions during monocyte-to-macrophage differentiation, as well as the alterations in chromosomal 3D structure during various disease occurrences. In B cells, we emphasized the concept of the I compartment and the roles of factors such as STAT3 and PTEN. In T cells, we discussed the impact of the loss of Tcf1 and SATB1 on the genome’s 3D structure. Finally, in neutrophils, we mainly elucidated the effects of the specific binding of the PU.1 factor on gene expression. Additionally, the use of deep learning-based Hi-C technology to study immune cells for diagnosing and treating diseases has emerged as a promising research area that has attracted considerable attention.

The Hi-C technique also has its significant limitations. Because Hi-C technology requires input from millions of cells to construct Hi-C libraries for analysis, studying populations of immune cells with limited numbers becomes challenging. Recently, researchers have developed Low-C, a method that only requires a small number of cells (approximately 1,000) as the starting material, and can ultimately generate high-resolution chromatin contact maps similar to Hi-C. Therefore, in the future, the technology for studying chromatin 3D conformation may shift towards methods that require less input material, which will aid in studying cell populations with limited numbers (Díaz et al., 2018). In addition, the complexity of the Hi-C experimental process may lead to compromised reproducibility and consistency of results, and the vast amount of Hi-C data requires more advanced tools for analysis and processing. Furthermore, the resolution of Hi-C may no longer meet the demands for studying finer genome structures, necessitating the development of higher resolution techniques to better reveal the intricate structure and interactions within the genome. We believe that in the future, more advanced three-dimensional chromatin structure technologies will emerge to overcome the limitations of Hi-C, providing higher resolution and a more comprehensive perspective, thereby delving deeper into the complex structure and function of the genome (Kong and Zhang, 2019).
